# Systemic Responses of Barley to the 3-hydroxy-decanoyl-homoserine Lactone Producing Plant Beneficial Endophyte *Acidovorax radicis* N35

**DOI:** 10.3389/fpls.2016.01868

**Published:** 2016-12-12

**Authors:** Shengcai Han, Dan Li, Eva Trost, Klaus F. Mayer, A. Corina Vlot, Werner Heller, Michael Schmid, Anton Hartmann, Michael Rothballer

**Affiliations:** ^1^Research Unit Microbe-Plant Interactions, Department Environmental Sciences, Helmholtz Zentrum München, German Research Center for Environmental Health (GmbH)Neuherberg, Germany; ^2^Research Unit Plant Genome and Systems Biology, Department Environmental Sciences, Helmholtz Zentrum München, German Research Center for Environmental Health (GmbH)Neuherberg, Germany; ^3^Department Environmental Sciences, Institute of Biochemical Plant Pathology, Helmholtz Zentrum München, German Research Center for Environmental Health (GmbH)Neuherberg, Germany

**Keywords:** *Acidovorax radicis*, 3-OH-C10-homoserine lactone, plant growth promoting bacteria (PGPB), systemic plant responses, flavonoids, endophytes

## Abstract

Quorum sensing auto-inducers of the *N*-acyl homoserine lactone (AHL) type produced by Gram-negative bacteria have different effects on plants including stimulation on root growth and/or priming or acquirement of systemic resistance in plants. In this communication the influence of AHL production of the plant growth promoting endophytic rhizosphere bacterium *Acidovorax radicis* N35 on barley seedlings was investigated. *A. radicis* N35 produces 3-hydroxy-C10-homoserine lactone (3-OH-C10-HSL) as the major AHL compound. To study the influence of this QS autoinducer on the interaction with barley, the *araI*-biosynthesis gene was deleted. The comparison of inoculation effects of the *A. radicis* N35 wild type and the *araI* mutant resulted in remarkable differences. While the N35 wild type colonized plant roots effectively in microcolonies, the *araI* mutant occurred at the root surface as single cells. Furthermore, in a mixed inoculum the wild type was much more prevalent in colonization than the *araI* mutant documenting that the *araI* mutation affected root colonization. Nevertheless, a significant plant growth promoting effect could be shown after inoculation of barley with the wild type and the *araI* mutant in soil after 2 months cultivation. While *A. radicis* N35 wild type showed only a very weak induction of early defense responses in plant RNA expression analysis, the *araI* mutant caused increased expression of flavonoid biosynthesis genes. This was corroborated by the accumulation of several flavonoid compounds such as saponarin and lutonarin in leaves of root inoculated barley seedlings. Thus, although the exact role of the flavonoids in this plant response is not clear yet, it can be concluded, that the synthesis of AHLs by *A. radicis* has implications on the perception by the host plant barley and thereby contributes to the establishment and function of the bacteria-plant interaction.

## Introduction

In the rhizosphere, microbes are selectively enriched as compared to the surrounding bulk soil due to the availability of plant root exudates. Plant growth promoting bacteria (PGPB) are part of this microbial community exhibiting beneficial effects on plants, like biocontrol activity toward plant pathogenic organisms and promotion of plant growth due to enhanced supply of limiting nutrients like phosphate, nitrogen and essential trace elements like ferric iron. Induced systemic resistance (ISR) caused by root associated bacteria enhances the defense even in foliar tissues for later pathogen attack (Lugtenberg and Kamilova, 2009). Many molecules, so-called MAMPS (microbial associated molecular patterns) including lipopolysaccharide (LPS), exopolysaccharide (EPS), and microbial flagella elicit ISR-responses (Berendsen et al., 2012). In addition, small secondary metabolites such as the siderophore pyoverdin, the antibiotics 2,4-DAPG and lipopeptides, pseudobactins, pyocyanin, and certain biosurfactants belong to the complex spectrum of elicitors of plant responses upon contact with microbes (De Vleesschauwer et al., 2008; De Vleesschauwer and Höfte, 2009; Guillaume et al., 2012; Chowdhury et al., 2015). Also volatile organic compounds, for instance 2R, 3R-butanediol, were shown to induce plant resistance (Cortes-Barco et al., 2010). PGPB cause ISR because they initiate a priming of specific initial plant responses, upon surface or endophytic colonization. The priming status includes no upregulation of pathogen related (PR) genes, which is required in systemic acquired resistance (SAR), known to be induced by plant pathogens. Upon additional specific stress situations, PR proteins were potentially activated (Pieterse et al., 1996; Ahn et al., 2007). Quorum sensing (QS) compounds of Gram-negative bacteria, like *N*-acyl homoserine lactones (AHL), were also found to cause systemic ISR-like responses in different plants (De Vleesschauwer et al., 2008; De Vleesschauwer and Höfte, 2009; Cortes-Barco et al., 2010). Compared to the already advanced knowledge on the responses of plants to a large number of systemic defense elicitors, details about the perception of plants regarding these QS signal molecules are still scarce, despite the importance these bacterial messenger molecules must have considering their presence throughout the entire plant evolution.

In many Gram-negative bacteria, *luxI*-*luxR* type quorum sensing (QS) systems use *N*-acyl-homoserine lactones (AHLs) as auto-inducing signals (Fuqua and Greenberg, 2002). The length of the acyl-residues of AHLs produced by the I-gene, varies from 4 to 18 carbon atoms and hydroxyl- or carbonyl-group substitutions are found at the C3-position. Most of these AHL signal compounds are able to diffuse through bacterial membranes freely, while specific transporters were found for AHLs with long chain fatty acid residues (Krol and Becker, 2014). Specific *luxR*-type receptors or transcription factors bind AHL signal molecules at elevated intracellular concentrations leading to increased expression of the *luxI-*gene. Subsequently, specific gene expression is activated or suppressed by binding and releasing the AHL-LuxR transcription factor from specific gene promoter regions. AHLs dependent QS circuits are global regulons; they control a wide range of biological functions including swarming motility, bioluminescence, plasmid conjugative transfer, biofilm formation, antibiotic biosynthesis, and the production of virulence factors in plant and animal pathogens (Eberl, 1999; Waters and Bassler, 2005). Since these AHL autoinducers also convey information about the surrounding and habitat quality of the cells, AHLs play a central role in optimizing the expression of their genetic repertoire and thus have an important efficiency optimizing function (Hense et al., 2007). It turned out that AHLs not only allow bacterial populations to interact with each other but are also recognized as signals by their eukaryotic hosts. C12- and C16- side chain AHL molecules are able to induce a specific and extensive proteome response in *Medicago truncatula* (Mathesius et al., 2003). Using *in situ* bioreporter bacteria for AHLs, a production of AHLs by *Serratia liquefaciens* MG1 and *Pseudomonas putida* IsoF colonizing the rhizoplane of tomato roots were demonstrated (Gantner et al., 2006). These strains exert beneficial effects on tomato plants when inoculated to roots, since it could be shown, that the ISR-like response toward the leaf attacking fungus *Alternaria alternata* was dependent on the production of C6-and C8-side chain AHLs by *S. liquefaciens* MG1 (Schuhegger et al., 2006). In contrast, in *Arabidopsis thaliana*, short side chain AHLs induced phytohormonal changes in the plants and an enhancement of root growth, but no priming of pathogen response (von Rad et al., 2008). In recent years, a series of specific perception responses in different plants were reported toward the addition of long-side chain AHLs and AHL producing bacteria to roots, as summarized by Schikora et al. (2016). While most of the effects of AHLs on plants were documented when AHLs were applied as pure compounds to the medium at the roots, much less is known about how AHL production by PGPB located on or inside the root contributes to the plant's perception of these bacteria. This is not only because root colonizers produce many other substances besides AHLs the plant will respond to, but also because due to the variable bacterial colonization pattern on the root surface, which ranges from microcolonies to dense biofilms. Therefore the AHL concentration will vary quite a lot locally, which is not well-reflected by the application of an average AHL concentration to the plant growth medium.

In plant response toward bacteria flavonoids play an important role. A high diversity of flavonoids are known in different plants (Hassan and Mathesius, 2012). In barley, the most abundant flavonoids are saponarin and lutonarin (Kamiyama and Shibamoto, 2012). Flavonoids contribute to biotic or abiotic stress resistance toward oxidative damage. They are known for their antioxidant, fungicide, bactericide, and anti-pest properties (Treutter, 2005; Cushnie and Lamb, 2011; Hassan and Mathesius, 2012). Flavonoid biosynthesis genes are expressed in a tightly regulated manner and include early flavonoid biosynthesis genes (EBG) like chalcone synthase (CHS; Hassett et al., 1999), chalcone-flavonone isomerase (CFI), 4-coumarate-CoA ligase (4-CL), and UDP-glucuronosyltransferase (UGT; Besseau et al., 2007). The AHLs 3-oxo-C12-HSL and 3-oxo-C14-HSL were found to induce several of these flavonoid synthesis genes (Mathesius et al., 2003; Schenk et al., 2014).

The model organism used in this study was the type strain N35 of *Acidovorax radicis*, an endophytic Gram-negative bacterium originally isolated from wheat roots (Li et al., 2011). In the genus *Acidovorax*, pathogenic as well as saprophytic or beneficial species are known. The majority of the *Acidovorax* spp. are phytopathogenic for diverse plants, but there are also ubiquitously distributed saprophytic environmental *Acidovorax* spp. in rhizosphere and water habitats, like *A. delafieldii, A. defluvii, A. temperans*, and *A. soli* which are more closely related to *A. radicis*. *A. radicis* N35 can colonize the surface and endosphere of barley roots and shows the ability to promote barley growth in soil under certain conditions. In its genome, a homologous *luxI-luxR* type gene pair was identified (Li et al., 2011). The *N*-acyl-homoserine lactone produced by *A. radicis* N35 was identified as *N*-(3-OH-C10)-homoserine lactone using high performance liquid chromatography and FT-ICR-mass spectrometry (Fekete et al., 2007).

The objective of this study was to investigate the effect of AHL production of *A. radicis* on root colonization and the perception by barley plants. Therefore, we compared the wild type strain N35 and an AHL negative mutant with disrupted *araI* gene in their influence on barley seedlings using RNA-sequencing of leaves of inoculated barley plants and q-PCR. The analysis was focused on the flavonoid biosynthesis as part of the defense response. The results indicated that the AHLs produced by *A. radicis* N35 reduced systemic defense responses like flavonoid accumulation in response to the colonization by this endophytic bacterium.

## Materials and methods

### Strains, culture media, and growth conditions

All strains and plasmids used in this study are listed in Table 1. *A. radicis* N35 was isolated from surface sterilized wheat roots (Li et al., 2011). It was grown in NB complex medium at 30°C at 180 rpm. Kanamycin (Km, 50 μg/ml) was supplemented to growth media of YFP-labeled *A. radicis* N35. The *A. radicis* N35 *araI* mutant was grown in NB medium containing 20 μg/ml tetracycline (Tc); for the GFP-labeled *A. radicis* N35 *araI* mutant, Km 50 μg/ml and Tc 20 μg/ml was added. *Agrobacterium tumefaciens* A136 (with plasmids pCF218 and pCF372) was cultured in NB medium with Tc 5 μg/ml.

**Table 1:** **Strains and plasmids**.

**Strains and plasmids**	**Relevant characteristics**	**Source**
*A. radicis* N35	Wild type	Li et al., 2011
*A. radicis* N35 YFP	Wild type, labeled with YFP, Km^R^	Li et al., 2012
*A. radicis* N35 *araI::tet*	AHL^−^ mutant, Tc^R^	This study
*A. radicis* N35 *araI::tet* C	AHL^−^ mutant, complemented with plasmid pBBR1MCS-2-AraI, Tc^R^, Km^R^	This study
*A. radicis* N35 *araI::tet* GFP	AHL^−^ mutant, GFP labeled with plasmid pBBR1MCS-2-GFP, Tc^R^, Km^R^	This study
*Agrobacterium tumefaciens* A136	AHL biosensor with pCF218, pCF372	Stickler et al., 1998
pBBR1MCS-2-GFP	GFP expression vector, Km^R^	Li et al., 2012
pBBR1MCS-2-YFP	YFP expression vector, Km^R^	This study
pBBR1MCS-2-AraI	*araI* gene expression vector, Km^R^	This study

For the inoculation of barley plants, 50 ml overnight culture of *A. radicis* N35 wild type and *araI* mutant strains were harvested using 4000 g by centrifugation (Eppendorf 5417R, Eppendorf, Hamburg, Germany) for 10 min at room temperature, and the supernatant was discarded. The cells were washed twice with 50 ml of 1x PBS and thereafter the cell concentration was adjusted to an optical density (OD_435nm_) of 1.5 (equal to 10^8^ cfu/ml) in 20 ml 1x PBS solution measured using a spectral photometer (CE3021, Cecil, Cambridge, England).

### Characterization of AHL production using AHL biosensor strain

AHL production of *A. radicis* N35 and its AHL deficient *araI* mutant were examined via a *traI-lacZ* fusion sensor plasmids in *A. tumefaciens* A136, which lacks the Ti plasmid and harbors the two plasmids pCF218 and pCF372. These two plasmids encode the *traR* and *traI-lacZ* fusion genes, respectively. These bio-reporter constructs allow highly efficient detection of AHLs (Stickler et al., 1998). The sensor strain was streaked to the center of an LB or NB agar plate containing 40 μg/ml X-gal, and the test bacterial strains were cross-streaked close to the biosensor. The culture plates were incubated at 30°C in the dark for 24–48 h. AHL production was detected via the activation of the reporter fusion *traI-lacZ*. In the presence of AHLs, beta-galactosidase activity was induced at the contact area of test and sensor strain. The metabolization of X-gal to the insoluble blue colored 5-bromo-4-chloro-3-hydroxyindole dimer indicates the presence of AHL molecules.

### Construction of an *araI* mutant strain

For knock-out mutagenesis in *A. radicis* N35, the *sacB* based gene replacement vector pEX18Gm described by Hoang et al. (1998) was used. First, a DNA cassette was constructed, which carried the *araI* target gene (amplified with primer pair AHLsyn-s2 GCCAGCTTGTCATAGGACTC and AHLsyn-as2 ATGCACCTCCAGAAAACG) disrupted by a Tc antibiotic marker (*tet* gene amplified with primer pair TcR-s AAAGTCTACTCAGGTCGAGG and TcR-as3 AAAGTAGACGACGAAAGGC). This cassette was cloned into the gene replacement vector pEX18Gm. Subsequently this constructed gene replacement plasmid was transferred into electrocompetent *A. radicis* N35 cells by electroporation. In the target cell a homologous recombination event occurred after pairing of the constructed DNA cassette with the homologous region in the genome of *A. radicis* N35, which led to an insertion of the whole constructed pEX18 plasmid into the genome of N35. The cells with integrated plasmid were selected on NB medium containing antibiotics. These merodiploids were resolved by plating on NB medium containing 5% sucrose, which led to cell death if the *sacB* gene was expressed. Only cells where the *sacB* gene together with the gentamycin selective marker was eliminated from the genome by a second homologous recombination could survive on sucrose containing medium. The resulting insertion mutants *A. radicis* N35 *araI::tet* carried a disrupted dysfunctional *araI* gene (Figure S1). The success of the knock-out mutagenesis was verified with PCR using the *araI* specific primers and by sequencing of the PCR products (ABI Prism, Applied Biosystems, Carlsbad, CA, USA). AHL production was then visualized by the *A. tumefaciens* A136 AHL biosensor as described above.

### Construction of fluorescence labeled *A. radicis* N35 and *araI* mutant strains

For YFP-labeling of *A. radicis* N35 *araI::tet*, plasmid pBBR1MCS-2-YFP, a YFP expressing broad-host range vector, and for GFP-labeling of the N35 wild type, plasmid pBBR1MCS-2-GFP were used. After isolation using a NucleoSpin plasmid kit (Macherey & Nagel, Düren, Germany) the plasmid was transferred to electro-competent cells of *A. radicis* N35 as described by Dower et al. (1988). Electroporation was performed with a Gene Pulser instrument (Bio-Rad, Munich, Germany) using a voltage of 2.5 kV for 4.5–5.5 ms. The resulting transformants were selected on Km containing NB plates and examined for specific fluorescence with an epifluorescence microscope at an excitation wavelength of 488 nm.

### Inoculation and growth of barley seedlings in axenic system

Before germination, seeds of barley (*Hordeum vulgare* L.) cultivar Barke were surface sterilized to eliminate microbial contaminations using the method described by Rothballer et al. (2008). The method was slightly modified by using antibiotics (streptomycin 250 μg/ml and penicillin 600 μg/ml) for 20 min before testing the seeds on NB plates for 2 days at 30°C in the dark for contaminations. After washing at least three times in sterilized water, 2 days old axenic barley seedlings with comparable root lengths of about 2 cm were selected for inoculation according to Li et al. (2012). Seeds were immersed in suspensions of *A. radicis* N35 and its derivative strains for 1 h before planting. For the inoculation with single bacterial strains or a bacterial mixture (v/v 1:1), the 2 days old barley seedlings were incubated in the bacterial suspension for 1 h at room temperature. Axenic cultivation of barley was performed in sealed and autoclaved glass tubes (3 cm width, 50 cm length, AG, Mainz, Germany) filled with 50 g autoclaved glass beads and 10 ml of sterile Murashige and Skoog mineral salt medium (Duchefa Biochemie, Haarlem, The Netherlands). The barley seedlings were grown at a 12 h photoperiod (metal halide lamps of 400 W) under a 23°C / 18°C day/night cycle for 10 days maximum until three-leaf stage in a growth chamber. The roots were harvested by taking the whole plant out of the glass tube, and rinsing off the adhering material with sterile 1x PBS.

### Visualization of fluorescence protein labeled bacteria

To visualize the GFP or YFP tagged *A. radicis* N35 colonizing barley roots, freshly harvested roots of barley were embedded in Citifluor and placed on glass slides. For each inoculation 6 root pieces of about 1 cm were observed. The fluorescence was detected using a confocal laser scanning microscope, CLSM 510 Meta (Zeiss, Oberkochen, Germany). The excitation wavelength at 488 nm was produced by an argon ion laser, the others at 543 and 633 nm by helium/neon lasers. Barley roots show auto-fluorescence which allows the visualization of the root structure. In the CLSM-images, roots were shown in magenta, GFP- labeled bacteria in green, and YFP-labeled bacteria in red color. CLSM lambda mode was used to discriminate between the very similar emission wavelengths of 510 nm for GFP and 530 nm for YFP (excitation for both 488 nm).

### Visualization of bacteria using fluorescence *in situ* hybridization

The FISH-method as described in Manz et al. (1992) and Amann et al. (1991) was applied modified for root samples as described in Rothballer et al. (2015). For *A. radicis* N35 the specific probe ACISP 145 (TTTCGCTCCGTTATCCCC), combined with an equimolar mixture of the universal bacterial probes EUB 338 I (GCTGCCTCCCGTAGGA), EUB 338 II (GCAGCCACCCGTAGGTGT), and EUB 338 III (GCTGCCACCCGTAGGTGT) were used. The specific fluorescence label was visualized by a CLSM using appropriate excitation wavelengths.

### Soil cultivation of barley and sample preparation

For the cultivation of barley in soil, commercial “Graberde” (nutrient limited substrate, Alpenflor, Weilheim, Germany) was mixed with sand (v/v 1:1). Each pot (10 cm height, 8 cm diameter) was filled with the same volume of soil substrate. One liter of tap water was added to initially water the pots. Barley seeds were germinated on paper towel by incubation at room temperature for 3 days (non-sterile conditions). Seedlings without inoculation were used as control. For bacterial inoculation, seeds were treated with cell suspensions of *A. radicis* N35 or the *A. radicis* N35 *araI* mutant (10^8^ cells ml^−1^ per seedling) for 1 h, as described above. In the plant growth promotion experiment for each treatment 15 pots with only one plant per pot were cultivated for 2 weeks or 2 months. The plants were watered twice a week. Throughout the experiment, the plants were fertilized once each week with Hoagland solution (10 ml 50x stock, diluted in 1 l water). Barley plants were grown under greenhouse conditions at temperatures of 15–25°C during the day and 10–15°C during the night.

For RNA-sequencing, q-PCR and HPLC analysis for each treatment four leaves of 2 weeks old barley seedlings, inoculated 10 days prior to harvest were pooled. At this time point under these cultivation conditions plants were in the three leave developmental stage and their height was <20 cm excluding roots. Always the second leaves were harvested. The leaves were shock frozen in liquid nitrogen and ground in a mortar resulting in about 150 mg of sample material. Fifty milligrams of this homogenate was transferred to a 2 ml Eppendorf tube and used for RNA or flavonoid extraction.

### RNA-sequencing

Total RNA was isolated from the prepared barley leave samples using RNeasy plant mini kit (QIAGEN, Hilden, Germany) according to the manufacturer's instruction. For each sample cDNA was generated using high capacity cDNA reverse transcription kit (Applied Biosystems, Carlsbad, CA, USA). cDNA-libraries were sequenced by the KFB Regensburg (Regensburg, Germany) using a HiSeq 2500 (Illumina, San Diego, CA, USA) in single-read mode running 100 cycles. Bioinformatic analysis was performed as described by Dugar et al. (2013). To ensure high sequence quality, Illumina reads in FASTQ format were trimmed with a cutoff phred score of 20 by the program fastq_quality_trimmer from FASTX-Toolkit version 0.0.13. The alignment of reads, coverage calculation, genewise read quantification, and differential gene expression were performed with READ emption which was relying on segemehl and DEseq version X (Hoffmann et al., 2009). Visual inspection of the coverages was performed using the integrated genome browser (IGB, http://bioviz.org/igb/index.html).

### q-PCR

Isolation of total RNA and generation of cDNA were performed as described above. Quantitative PCR (q-PCR) was performed using the primers as listed in Table 2 and the SYBR green kit (PeQSTAR) of the real-time PCR system (PeQSTAR SEQ, VWR, Darmstadt, Germany). All primer pairs were verified by melting curves showing only one peak and a slope value close to −3.33. Transcript accumulation was analyzed using relative quantification with the software sigma plot. The q-PCR results are the average of three technical repetitions per sample and five independent plant inoculation experiments.

**Table 2:** **Primer used in qPCR-analysis**.

**Genes**	**Primer name**	**Slope value**	**Primer sequence**	**Gene name**
MLOC_67149	Q1	−3.48	AAGGCATGGGAGATGGTTGG	F-Box family-3(fb-3)
			TATCATGGCGTCCCACACG	
MLOC_10956	Q20	−3.62	GCCAGAAGCCATATCTGCAC	UDP-glycosyltransferase-like protein (UGT)
			GCAGAAAAACTCACCGGAGC	
MLOC_58764	Q29	−3.35	TGACACCCCTGCTTCGTTAG	4-coumarate:CoA ligase (4-CL)
			ACGACAGCGACCTGTGTTAG	
MLOC_5324	Q30	−3.74	CTTCGACGCACTTGTCTCGG	Chalcone-flavonone isomerase (CFI)
			ACTGCGACCCCTTGATCTCC	
MLOC_74116	Q32	−3.87	CCGACTACCCGGACTACTAC	Chalcone synthase (CHS)
			TGTACCTCTTCCTGATCTGCG	
MLOC_72837	Q24	−3.33	TGCTGCACAACTTTCACTCC	Chaperone protein (DnaJ)
			ACTGAAACTCCCATCCCAGC	
MLOC_59602	Q13	−3.25	ACTGAAACTCCCATCCCAGC	E3 ubiquitin-protein ligase (PRT1)
			TAGACCCTCCGCTGGTATCC	

### HPLC quantification of flavone glycosides in barley leaves

Ten microliters of methanol (HPLC-grade) was added for every mg of frozen sample material prepared as described above. The samples were then vortexed for 1 h on a lab shaker at 700 rpm in the dark. Samples were then centrifuged for 10 min at 11,000 rpm. The supernatants were transferred to a new cap and stored until HPLC-measurement at −80°C. For HPLC analysis, a reversed-phase HPLC system was applied. A linear gradient over 45 min was applied with 100% solution A (2% formic acid containing 0.1% ammonium formate) to 100% solution B (0.1% ammonium formate in 88% methanol) and maintained for another 5 min. Finally, the absorbance of the eluent was measured at 280 nm (Yin et al., 2012).

## Results

### Construction of AHL synthase mutant and *araI* complemented strain

**Figure 1:**
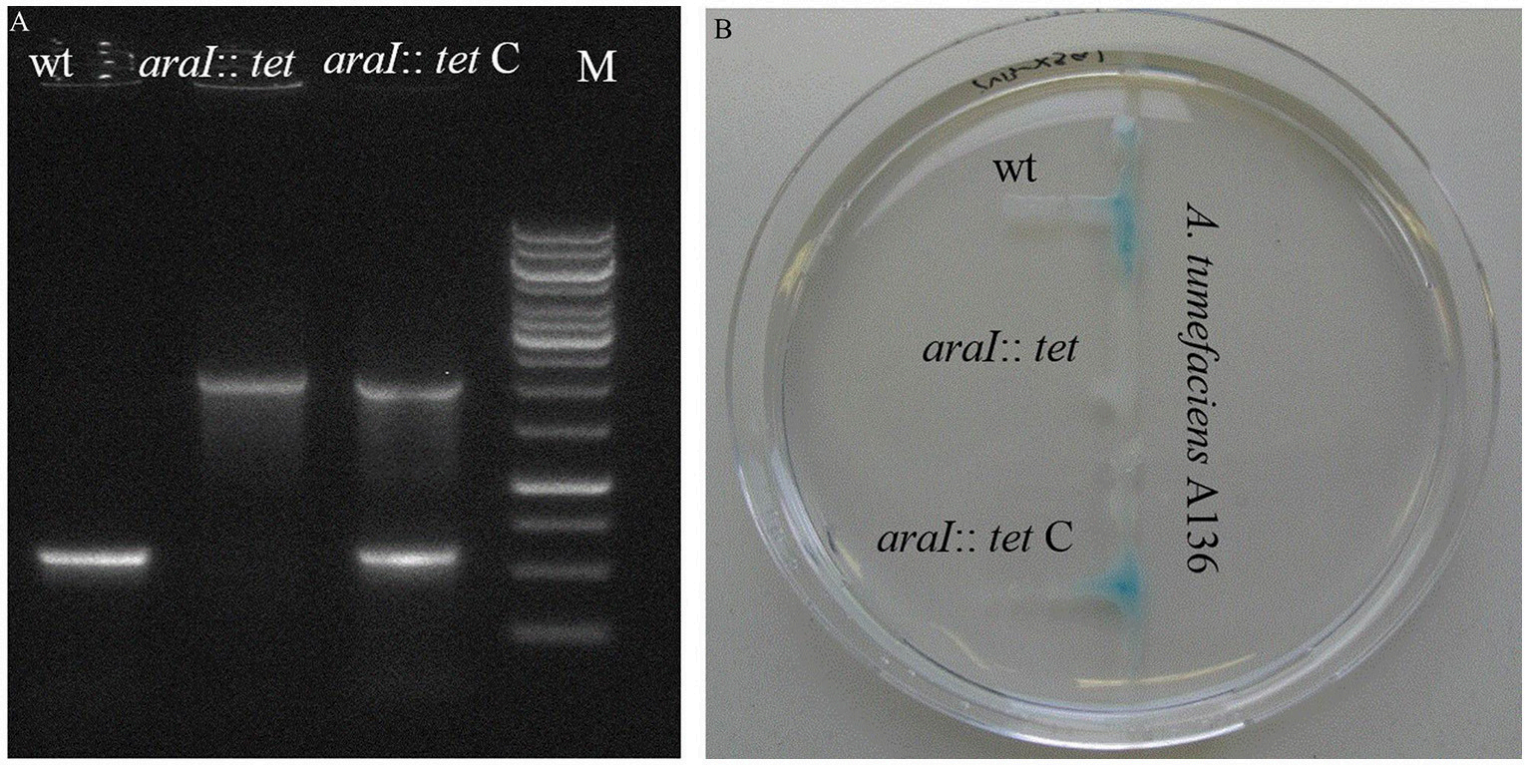
**PCR verification (A)** and a galactosidase biosensor plate assay detecting AHL production **(B)** of the *A. radicis* N35 wildtype, *araI* knock-out mutant (*araI::tet*), and complemented *araI* knock-out mutant (*araI::tet* C).

To investigate the QS function in *A. radicis* N35, an AHL deficient mutant was constructed by disrupting the AI synthase gene *araI* with a tetracycline resistance gene (1.5 Kb). A complementary strain was produced by cloning the *A. radicis* wild type *araI* gene into the broad host range vector pBBR1MCS-2 and transferring this construct into the *araI* knock-out strain. PCR amplification with *araI* specific primers using as a template a complete DNA extract (containing genomic and plasmid DNA) from the *araI* mutant and complemented mutant resulted in the expected band sizes (Figure 1A). Subsequent sequencing verified the identity of the PCR amplificates and correct construction of the knock-out cassette and complementary plasmid. To test the AHL production, the AHL biosensor *A. tumefaciens* A136 (carrying pCF218 or pCF372) was applied. This strain can detect various types of AHLs, especially C10-HSL including the hydroxyl- or oxy-derivative at position C3 (Stickler et al., 1998). Figure 1B shows AHL production indicated by the blue color only for the wild type and the complemented *araI* mutant, and not for the uncomplemented mutant, which proves the successful knock-out of the *araI* gene.

### Colonization of roots using differentially fluorescence labeled wild type and *araI* mutant strains

**Figure 2:**
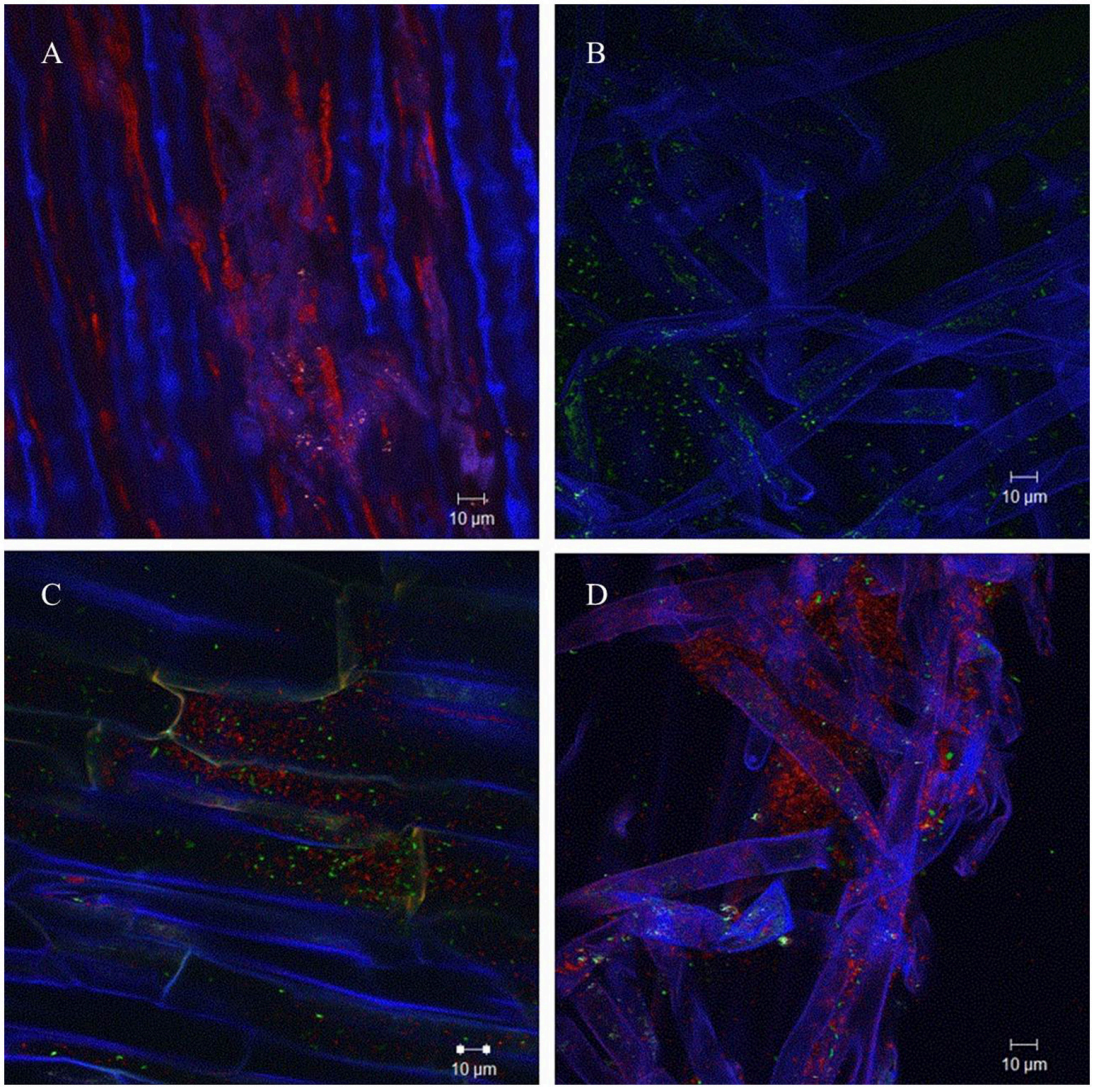
**Colonization of barley roots by fluorescence labeled *A*. *radicis* N35 wild type and *araI* mutant detected using CLSM lambda mode**. Blue color represent barley roots, red color represent the YFP-labeled *A. radicis* N35 wild type and green color represents the GFP labeled *A. radicis* N35 *araI::tet* AHL mutant. **(A)** YFP-labeled *A. radicis* N35 forms biofilm in the main root part and root hair part. **(B)** GFP-labeled *A. radicis* N35 *araI::tet*. **(C,D)** Inoculation with GFP-labeled *A. radicis* N35 *araI::tet* mutant mixed 1:1 with YFP-labeled *A. radicis* N35 wild type.

In order to analyze if AHL production of *A. radicis* N35 had an influence on the colonization ability on barley roots, differentially GFP/YFP-labeled wild type and *araI* mutant strains were applied. The differentially fluorescence labeled bacteria were applied to barley roots separately as well as in equal mixtures, and the barley seedlings were cultivated under axenic conditions for 1 week. After harvesting and washing the roots, the colonization behavior of wild type and *araI* mutant on the roots was examined using a CLSM in lambda mode, which allows to distinguish the fluorescence of GFP and YFP based on their specific emission spectrum. Both wild type and *araI* mutant colonized barley roots well when applied separately, although the *araI* mutant showed more of a single cell colonization pattern and less microcolony formation than the wild type. In general, most bacteria were found to colonize the basal parts of roots especially in the root hairs and the branching sites of side roots. When the *A. radicis* N35 wild type and *araI* mutant were applied in a 1:1 mixed inoculum, the wild type clearly dominated in colonization over the AHL negative strain (Figure 2). This indicates that AHL production by *A. radicis* N35 is important for its competitive colonization ability on barley roots.

### Plant growth promotion effect

**Figure 3:**
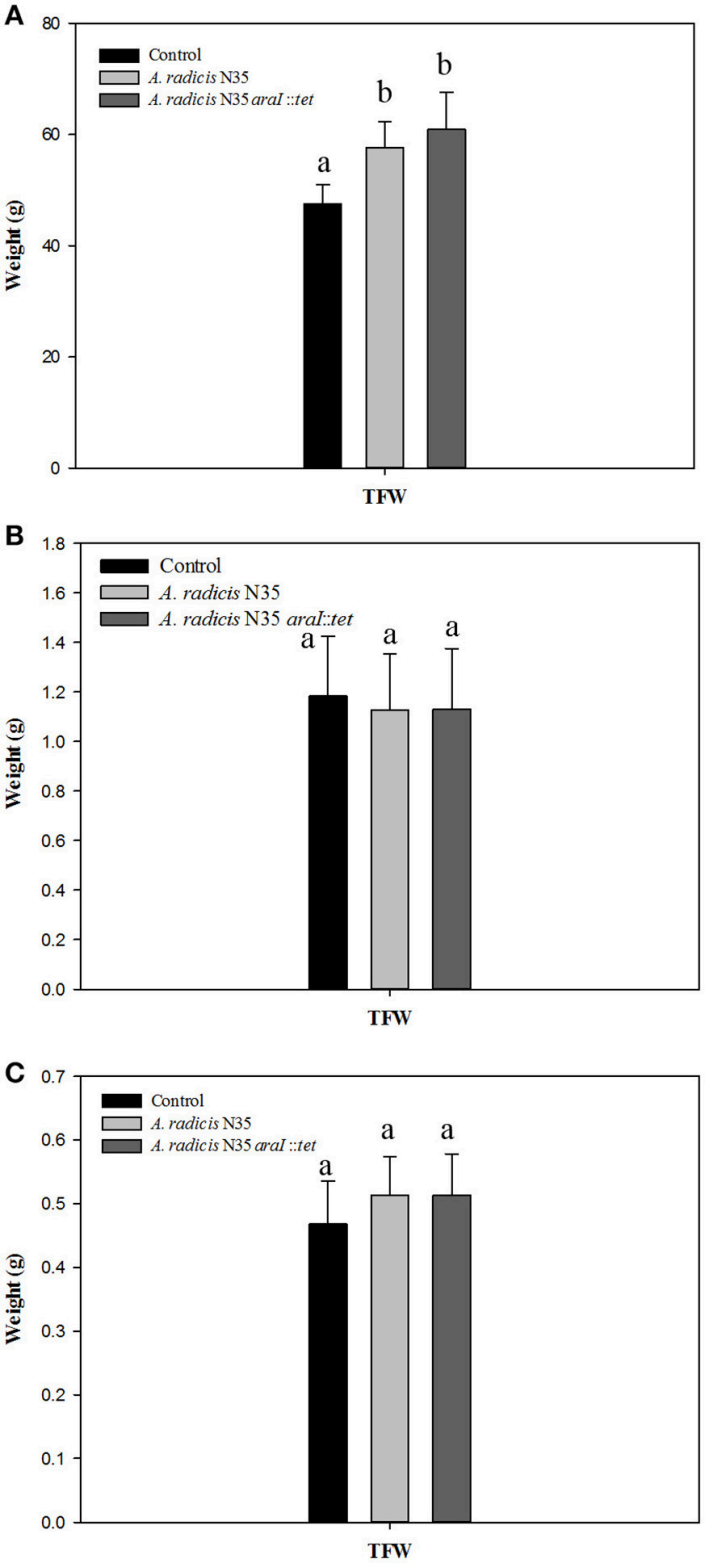
**(A,B)** Total fresh weights (TFW) of barley seedlings grown in soil after inoculation with *A. radicis* N35 wild type or the *araI* mutant after 2 months and 2 weeks, respectively. **(C)** Total fresh weight of barley seedlings 2 weeks after inoculation with *A. radicis* N35 wild type or the *araI* mutant, respectively, under axenic growth condition. Different letters indicate significant differences among treatment groups (*p* < 0.05).

To assess whether a growth promoting effect of *A. radicis* was possibly dependent on AHL production, seedlings were inoculated with N35 wild type and the *araI* mutant strains or not inoculated as control and grown under axenic conditions in the growth chamber or in non-sterile soil in the greenhouse. After 2 weeks or 2 months in the soil system and 2 weeks in the axenic system, barley plants were harvested and total fresh weight of the plants was measured (Figure 3). In the soil system, a significant growth promotion effect in response to inoculation with *A. radicis* N35 and the *araI* mutant on total plant fresh weight was found after 2 months (Figure 3A). In the axenic system no significant stimulation of growth was detectable after 2 weeks upon inoculation (Figure 3C). When the colonization of roots was analyzed using FISH, only a few cells of *A. radicis* N35 could be detected after 2 weeks, and it was not detectable anymore after 2 months in the soil system (not shown). In the axenic system, the colonization by *A. radicis* N35 was very well detectable with FISH (not shown) and GFP labeled cells during the whole growth period of 2 weeks (Figure 2 and Figure S2).

### Barley transcriptome analysis

**Figure 4:**
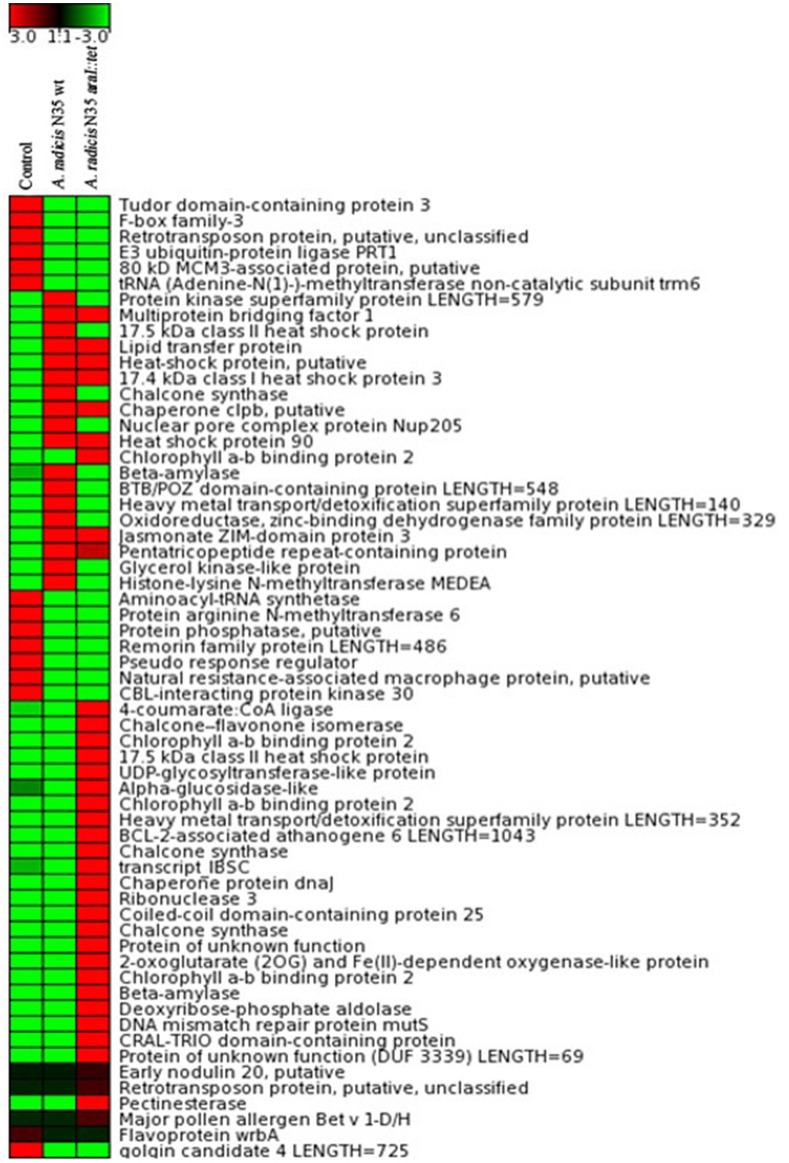
**Transcriptome analysis by RNA sequencing from 4 pooled leaves of barley plants grown in parallel**. Total RNA was isolated from barley leaves 10 days after root inoculation with *A. radicis* N35 wild type and the *araI* mutant, respectively. Red (upregulated) and green (downregulated) colors represent an at least three-fold difference in the amount of detected gene transcripts for the respective gene between the analyzed samples. Black color means no change between the expression levels of the found transcripts.

**Figure 5:**
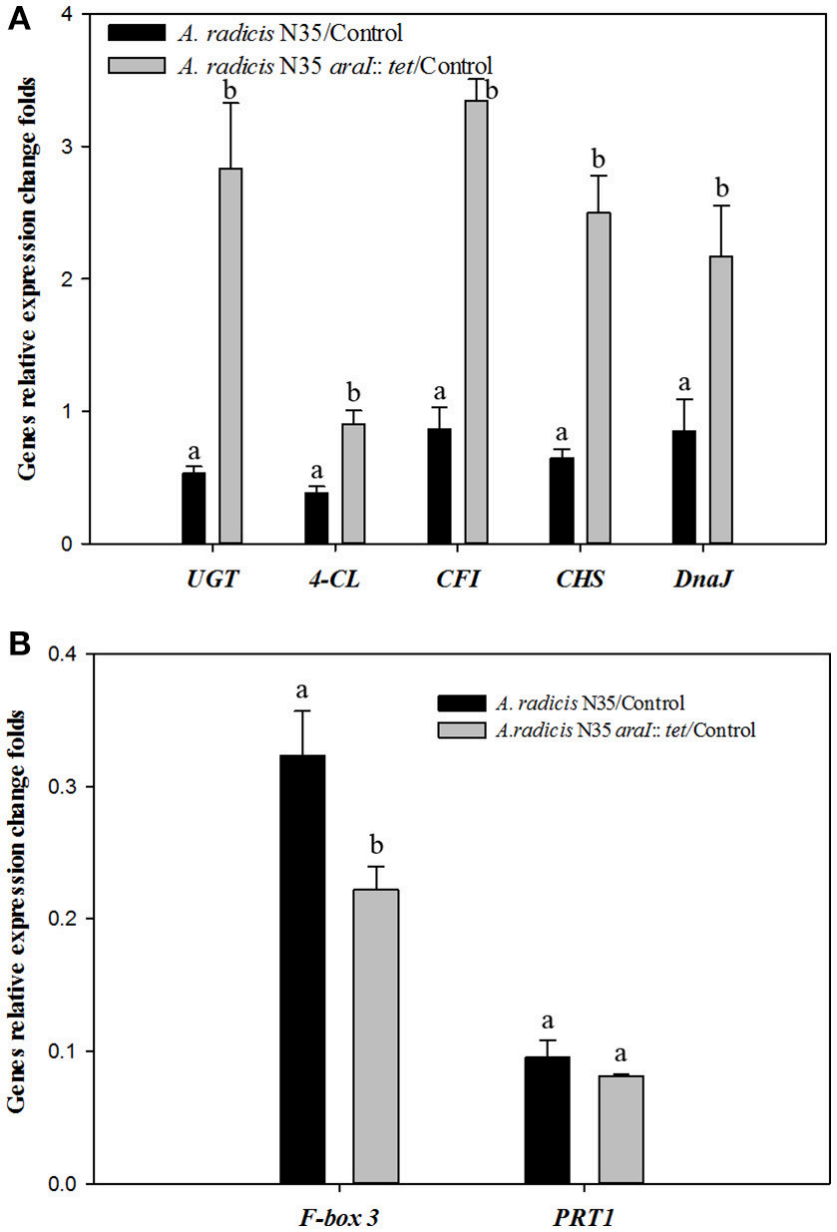
**q-PCR analysis of the expression of genes under the influence of *A*. *radicis* N35 wild type and the *araI* mutant**. Barley seedlings were not inoculated (control, CK) or inoculated with *A. radicis* N35 wild type or the *araI* mutant, respectively. Cultivation was performed monoxenically for 10 days (see MM). Then one leaf was taken at the three leaves stage, RNA was isolated, q-PCR from transcribed cDNA was performed. Statistical analysis was performed using one way ANOVA (*p* ≤ 0.05). Different letters indicate significant differences among treatment groups. **(A)** Flavonoid biosynthesis pathway genes: 4-coumarate CoA ligase (4-CL), chalcone-flavonone isomerase (CFI), chalcone synthase (CHS), UDP-glycosyltransferase-like protein (UGT); and chaperone protein (DnaJ). **(B)** Fb-3, F-Box family-3 and E3 ubiquitin-protein ligase (PRT1).

To investigate which plant genes were differentially regulated in barley leaves after inoculation in response to colonization by AHL producing and non-producing *A. radicis* N35 compared to the un-inoculated control plants, the plant transcriptome was analyzed via next generation sequencing alongside with a series of specific qPCR assays for verification of the sequencing results. In the barley leaf transcriptome sequencing analysis a number of gene transcripts were found to be significantly enhanced or suppressed by *A. radicis* N35 and/or the *araI* mutant at 10 days post inoculation (dpi) compared to the un-inoculated control plant (Figure 4). These plant transcripts can be classified into two groups: (1) AHL independent transcripts, correlated to the presence or absence of *A. radicis* N35, regardless if wild type or mutant were inoculated, and (2) AHL dependent transcripts, correlated to whether or not inoculated *A. radicis* N35 was able to produce AHL. Interestingly, transcript sequencing results from leaves after 10 dpi indicated that the transcription of several flavonoid synthesis genes (Besseau et al., 2007) was upregulated when plants were inoculated with the *araI* mutant (transcript group 2), including UDP-glycosyltransferase-like protein (UGT), CFI, chalcone synthase (CS), 4-coumarate-CoA ligase (4-CL) and chaperone protein (DnaJ). Thus, these five transcripts were selected for verifying the results by qPCR. Additionally, two genes of transcript group 1 were also selected, namely F-box family-3 gene (fb-3, 1) and the E3 ubiquitin-protein ligase PRT1 gene (PRT1), which were downregulated in response to inoculation with *A. radicis* N35 compared to the uninoculated control. Primers were designed for each of these seven genes (Table 2) and tested based on the standard and melting curve (Figure S4). The q-PCR assays (Figure 5) confirmed the transcriptomic sequencing data for the five group 2 transcripts related to the flavonoid pathway. The expression of these genes was between two- and four-fold higher with the *A. radicis* N35 *araI* mutant than with the AHL producing wild type. The downregulation of the two group 1 genes by both mutant and wild type could also be confirmed.

### Content of saponarin and lutonarin in barley leaves

**Figure 6:**
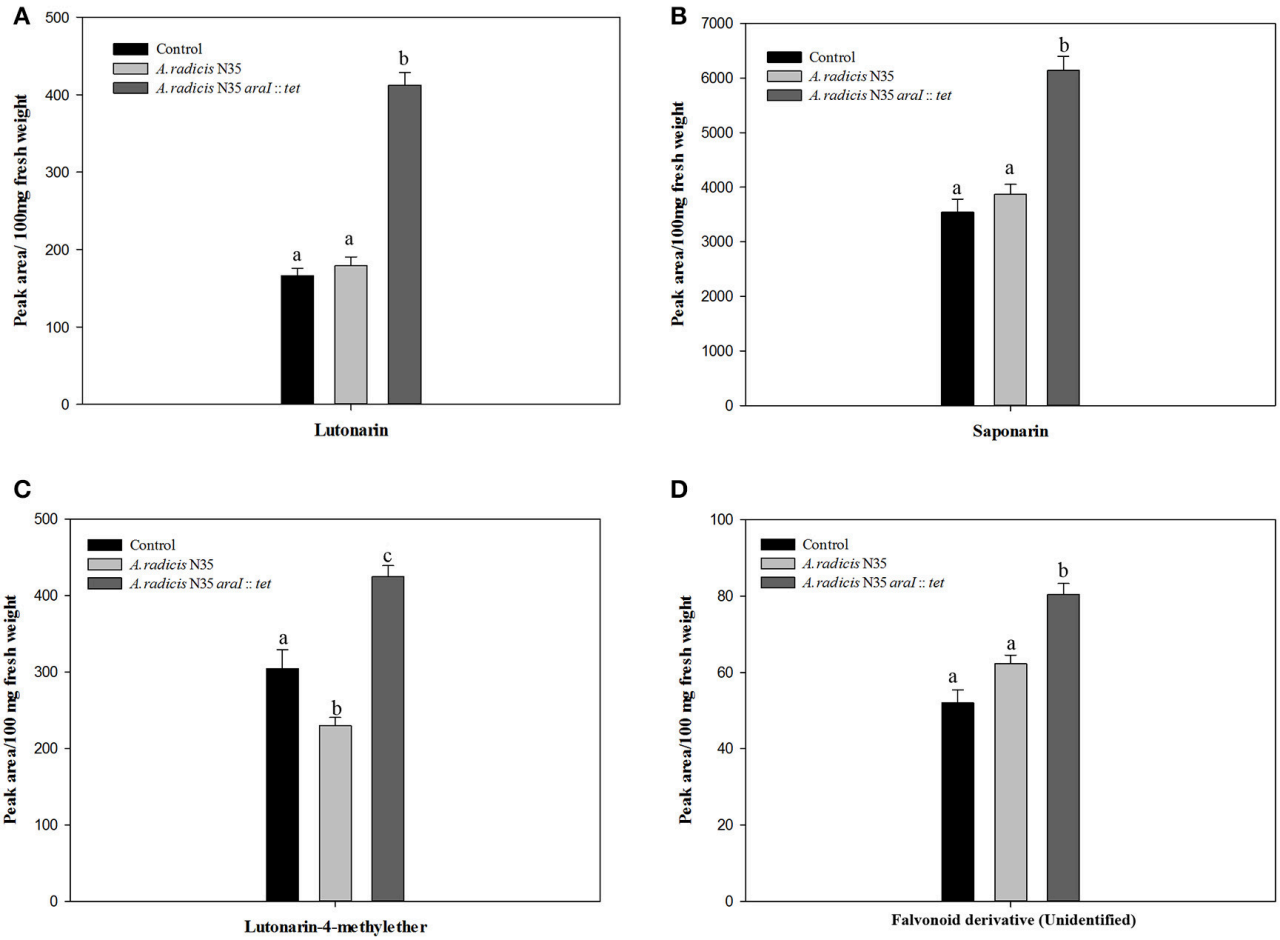
**Accumulation of flavonoids in barley leaves measured by HPLC**. The flavonoid components are **(A)** lutonarin, **(B)** saponarin, **(C)** lutonarin-4-methylester, **(D)** an unidentified flavonoid derivative. Different letters indicate significant differences among treatment groups (*p* < 0.05).

The HPLC-analysis revealed that in the leaves of the tested barley cultivar Barke the concentration of saponarin was generally higher than of lutonarin. In plants inoculated with the *A. radicis* N35 *araI* mutant, the contents of saponarin and lutonarin were ~two-fold compared to plants inoculated with the wild type or non-inoculated controls (Figure 6 and Figure S3). In addition the amount of lutonarin methylether in *araI* mutant inoculated plants reached almost twice the level detectable in wild type inoculated and non-inoculated control plants. These results corroborate the data from the gene transcriptome profiling by sequencing and q-PCR, which leads toward the conclusion that AHL signaling by barley root colonizing *A. radicis* N35 impacts flavonoid production in barley leaves.

## Discussion

### Influence of AHLs on the root colonization of *A. radicis* N35

AHL production by *A. radicis* N35 has a positive effect on the colonization of roots, since an AHL defective mutant was less successful in root colonization (Figure 3). The QS-deficient *araI* mutant strain showed colonization mostly by single cells spread randomly over the root surface, while the N35 wild type cells grew more aggregated in microcolonies. This result corroborates the observation by Li (2010), who showed in a 1:1-mixture of GFP-labeled N35 wild type cells and the SYTO orange labeled *araI* mutant that only a few mutants colonized the roots, while wild type cells showed dense colonization. Even though the mutants were colonized together with the wild type in these experiments, the AHL deficient cells could obviously not profit from the produced AHL by the wild type in their vicinity. However, it has been shown previously (Hense et al., 2007), that quorum sensing on the rhizoplane must be considered as a strictly localized phenomenon, mainly taking place in microcolonies forming in small niches on the root surface confined by diffusion limitation. Here, the signaling substances can locally reach very high concentrations far above the average values which are measurable by taking e.g., liquid samples from the rhizosphere. Thus, although some mutant cells might profit from AHL producing neighboring wild type cells, the overall AHL concentration these mutants were exposed to, was probably below the threshold levels required for the autoinduction process. Consequently, QS regulated genes were not activated which might have led to the observed low competitive colonization phenotype.

In *A. radicis* N35, also phenotypic variants showed reduced root colonization. However, in contrast to *araI* mutants these variants had much reduced ability of plant growth promotion (Li et al., 2012). The reduced colonization of the *araI* mutant could be caused by a reduced tolerance toward reactive oxygen species (ROS) released by barley roots upon first contact with microbes as has been found in the case of the endophyte *Gluconacetobacter diazotrophicus* during colonization of rice roots (Alquéres et al., 2013). In this case, ROS-quenching enzymes catalase and superoxide dismutase of the endophyte have a major role in the degradation of ROS released by the host plants during early host defense. In *P. aeruginosa*, QS was found to be involved in the stress tolerance, and *luxI-*type QS-deficient mutants (*lasI, rhlI*, and *lasI rhlI*) have defective expression of catalase (CAT) and superoxide dismutase activities (SOD). These mutants were more sensitive to oxidative stress than the parental strain (Hassett et al., 1999). Another study showed that the QS based stress tolerance can make it more difficult to quench quorum sensing activities and help to prevent social cheating (García-Contreras et al., 2015). In the co-inoculation experiment, the quorum sensing *araI* mutant may behave even as such a quorum sensing cheater, because it does not produce any AHL. The positive influence of QS on biofilm formation was shown in several studies. For instance, in the *Pseudomonas fluorescence* 2p24, the *pcoI* coded AHL synthase mutant resulted in seriously decreased biofilm formation, leading to less root colonization ability (Wei and Zhang, 2006). In *P. aeruginosa*, a quorum sensing *lasI* mutant formed flat undifferentiated biofilms which are more sensitive to the biocide sodium dodecyl sulfate than the wild type. These flat biofilm types of AHL defective mutants could be restored by exogenous addition of AHLs (Davies et al., 1998). Also in *Sinorhizobium fredii* SMH12, micro-colony biofilm formation was found regulated by QS and in *Rhizobium* spp., the biofilm formation is dependent on the production of AHLs (Davies et al., 1998; Rinaudi and Giordano, 2010; Pérez-Montaño et al., 2014). It could be shown, that exogenous addition of AHLs could promote the biofilm formation by *Acidovorax* sp. strain MR-S7 (Kusada et al., 2014). The importance of QS for the biofilm formation could be due to secretion of important compounds like extracellular DNA, the biosurfactant rhamnolipid and the secretion of the BapA-protein as shown in *P. aeruginosa* (Tolker-Nielsen, 2015). Furthermore, QS-compounds play an important role in *P. fluorescence* 2p24 for its colonization on wheat roots and development of biocontrol ability toward the take-all disease fungus (Wei and Zhang, 2006). In *Burkholderia phytofirmans* PsJN QS was also found to be important for its competitive biofilm formation and efficient colonization of roots and beneficial interaction with *A. thaliana* as plant host (Zúñiga et al., 2013). Thus, there is an increasing knowledge about the important role of AHLs in plant beneficial rhizosphere bacteria and endophytes in different plant systems and their involvement in different mechanisms of plant growth promotion. It could be even shown, that the exopolysaccharide production in *S. fredii* NGR234 was modified by AHL production (Krysciak et al., 2014).

### Influence of AHL producing *A. radicis* on the biosynthesis and content of flavonoids in barley leaves

Compared with *A. radicis* N35 wild type bacteria, the colonization of roots by the AHL deficient *araI* mutant caused an accumulation of saponarin and lutonarin in barley leaves (Figure 6). This indicates that AHLs themselves or bacterial components induced by AHLs are involved in the regulation / induction of flavonoid biosynthesis in the host plant. A direct stimulatory effect of AHLs on the induction of flavonoid biosynthesis was first found in *M. truncatula*. In this case, 3-oxo-C12-HSL was shown to activate the transcription of chalcone synthase genes in white clover roots (Mathesius et al., 2003). In the *A. radicis* N35—barley interaction, a different AHL (3-OH-C10-HSL) is operating, which may have caused an inhibition of flavonoid biosynthesis. In *A. thaliana*, the influence of the length of the acyl chain and the substitution at the AHL C3 position were shown to cause different systemic responses (Schikora et al., 2011). The contrasting response of barley to 3-OH-C10 HSL may also be due to the fact that the monocotyledonous barley may respond differently to AHLs than the dicotyledonous white clover. On the other hand, since QS autoinducers are able to regulate bacterial surface exopolysaccharide production (Krysciak et al., 2014), the lack of AHLs in the *A. radicis araI* mutant could also have resulted in considerable changes in the surface exopolysaccharide structure and this may have caused a different plant response.

Flavonoids are known to help plants to acquire resistance toward various biotic and abiotic stresses (Treutter, 2005). For example, flavonoids were found in relation to drought stress in winter wheat (Ma et al., 2014) and could exhibit antifungal activities, e.g., in the carnation-*Fusarium* pathosystem (Curir et al., 2004). Furthermore, lutonarin and saponarin isolated from barley sprouts have been shown to be effective against bacterial pathogenesis enzymes (Park et al., 2014). Finally both lutonarin and saponarin are frequently discussed as antioxidative and antimicrobial agents in alternative medicine, as e.g., recently reviewed in Lahouar et al. (2015). The enhanced accumulation of the two flavone glycosides saponarin and lutonarin in barley leaves caused by the colonization of the roots by the *A. radicis* N35 *araI* mutant is an example for this kind of defense response. The expression of several flavonoid biosynthesis genes was upregulated due to inoculation with the *A. radicis* N35 *araI* mutant. This clearly indicated that the AHL deficient mutant strains activated a defense response. Three closely related R2R3-MYBs transcription factors (MYB11, MYB12, and MYB111) redundantly activate the transcription of early flavonoid biosynthesis genes (EBGs). The UDP-glycosyltransferases UGT91A1 and UGT84A1 together with CHS, CHI, and F3H, FLS1 are controlled by this R2R3MYB factors in Arabidopsis (Stracke et al., 2007). However, no data were obtained for the regulation at the transcriptional level. The flavonoid accumulation is not only regulated at the transcriptional, but also at the post-transcriptional level through PAL-degradation mediated by Kelch domain-containing F-box (KFB) proteins and degradation of E3 ubiquitin ligase (PRT1) complexes leading to the suppression of the phenylpropanoid pathway (Feder et al., 2015). In this study, it could be demonstrated by the transcriptomic sequencing results and confirmed by q-PCR, that the expression of F-box protein and E3 ubiquitin-protein ligase were downregulated (Figure 5). F-box family proteins are components of the SCF-protein complex, which is involved in the proteome degradation pathway. This process is for example important for plant development and immunity response to various stress condition (Thines et al., 2007). In addition, also an upregulation of *dnaJ* expression after inoculation with the *araI* mutant was shown mediated by transcription factor SG7 MYB (Figure 5). Its expression was found to correlate with flavonoid related genes and to be under the control of MYB transcription factors (Stracke et al., 2007). The upregulation of *dnaJ* expression also correlated with the upregulation of the flavonoid biosynthesis genes and flavonoid accumulation. DnaJ is also involved in salt stress resistance and known to interact with Hsp70 in the heat shock resistance process (Zhu et al., 1993). Since *dnaJ* expression was also found to be involved in regulation of saline tolerance, it is reasonable to test whether the higher expression of *dnaJ* will also result in increased salt stress tolerance by the plants.

### Integrated role of AHLs by *A. radicis* in plant perception

Due to common evolutionary history, plants, and microbes have developed an elaborate system of mutual detection, cooperation, or deterrence. In the first recognition step, the most important function is the plants' innate immune system recognizing MAMPS and diverse microbial elicitors. On the microbial side the response to plant surface structures and exudates have a central role in recognition. The quorum sensing communication system of rhizobacteria based on AHL compounds may be considered as an integrated part in the perception of bacteria by plants. In the plant growth beneficial endophyte *A. radicis* N35, 3-OH-C10-HSL is the dominant AHL (Fekete et al., 2007). However, many Gram-negative plant pathogenic rhizobacteria also synthesize AHLs, although with different chain lengths and other functional groups. Since the onset of virulence is regulated by these auto-inducers, the plant is expected to learn about the presence of AHLs in their vicinity as soon as possible. In the case of pathogens, the network of multiple interactions concludes to initiate full expression of the defense cascade, while in the case of beneficial endophytes the response is dampened or completely suppressed allowing a cooperative interaction. There are several examples, that AHL compounds applied to rooting solutions of plants can exert diverse beneficial effects on plants, which include growth promotion as well as priming or induction of pathogen resistance in the host. This was shown in different plant species, such as *M. truncatula*, tomato, *A. thaliana*, and barley (Mathesius et al., 2003; Schuhegger et al., 2006; von Rad et al., 2008; Schenk et al., 2014). However, it is much less clear, what role AHLs of a beneficial root colonizing bacterium play in the concert of interaction with all its other compounds in the plant's recognition and perception system. In the current study, it could be shown, that the production of 3-OH-C10-HSL during the colonization process by the plant growth promoting, endophytic *A. radicis* N35 is able to efficiently influence the plant response and reduce the onset of a defense cascade. Whether this is caused by a direct interaction with AHLs or by an indirect effect through the induction of e.g., a different surface structure of the bacteria in the presence of AHLs, which is not recognized as a pathogenic signal, is not known yet. Nevertheless, the response of barley plants to *A. radicis* N35 wild type is characterized by the absence of expression of several genes involved in flavonoid biosynthesis in the plant leading to an attenuated defense response. Apparently, the 3-OH-C10-HSL-production is playing a major role in this process. Future detailed studies need to focus on the role of the quorum sensing compound 3-OH-C10-HSL on the regulation of expression of enzymes involved in the modification of the fine structure of the cell surface lipo- and exopolysaccharides or of type III secretion systems or other transport systems potentially involved in bacteria-host interactions.

## Author contributions

SH and DL performed the experimental work and wrote the paper; ET and KM did bioinformatic data analysis; AV and WH did plant metabolite analysis and discussion; MS, AH, and MR conceived the research and wrote the paper.

## Funding

This study was funded by the China Scholarship Council (CSC), file No. 2011911941 to SH.

### Conflict of interest statement

The authors declare that the research was conducted in the absence of any commercial or financial relationships that could be construed as a potential conflict of interest.

## Supplementary material

Figure S1 **Construction of *araI* mutant in *A. radicis* N35**.

Figure S2 **Colonization of barley roots by *A. radicis* N35 GFP in a monoxenic system at different time points after inoculation (dpi as indicated in the pictures)**.

Figure S3 **Upper part: HPLC-separation of compounds with absorbance at 280 nm (see MM)**. Lower part: UV-spectra of the different eluted compounds.

Figure S4 **qPCR primer melting curves**.
