# Supplementary figures and images for: Systemic Responses of Barley to the 3-hydroxy-decanoyl-homoserine Lactone Producing Plant Beneficial Endophyte Acidovorax radicis N35
Source: Front Plant Sci. 2016 Dec 12;7:1868. doi: 10.3389/fpls.2016.01868 (PMC5149536; doi:10.3389/fpls.2016.01868)

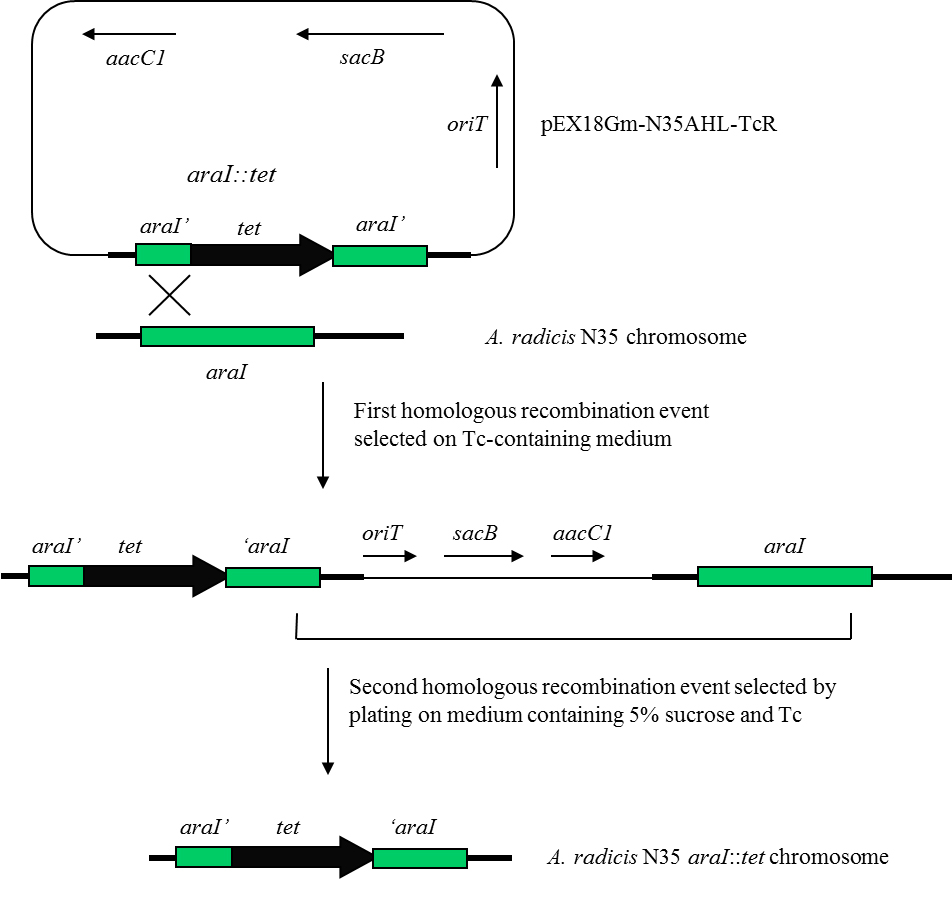

Supplement: Figure S1 — Construction of araI mutant in A. radicis N35. [file Image1.JPEG]

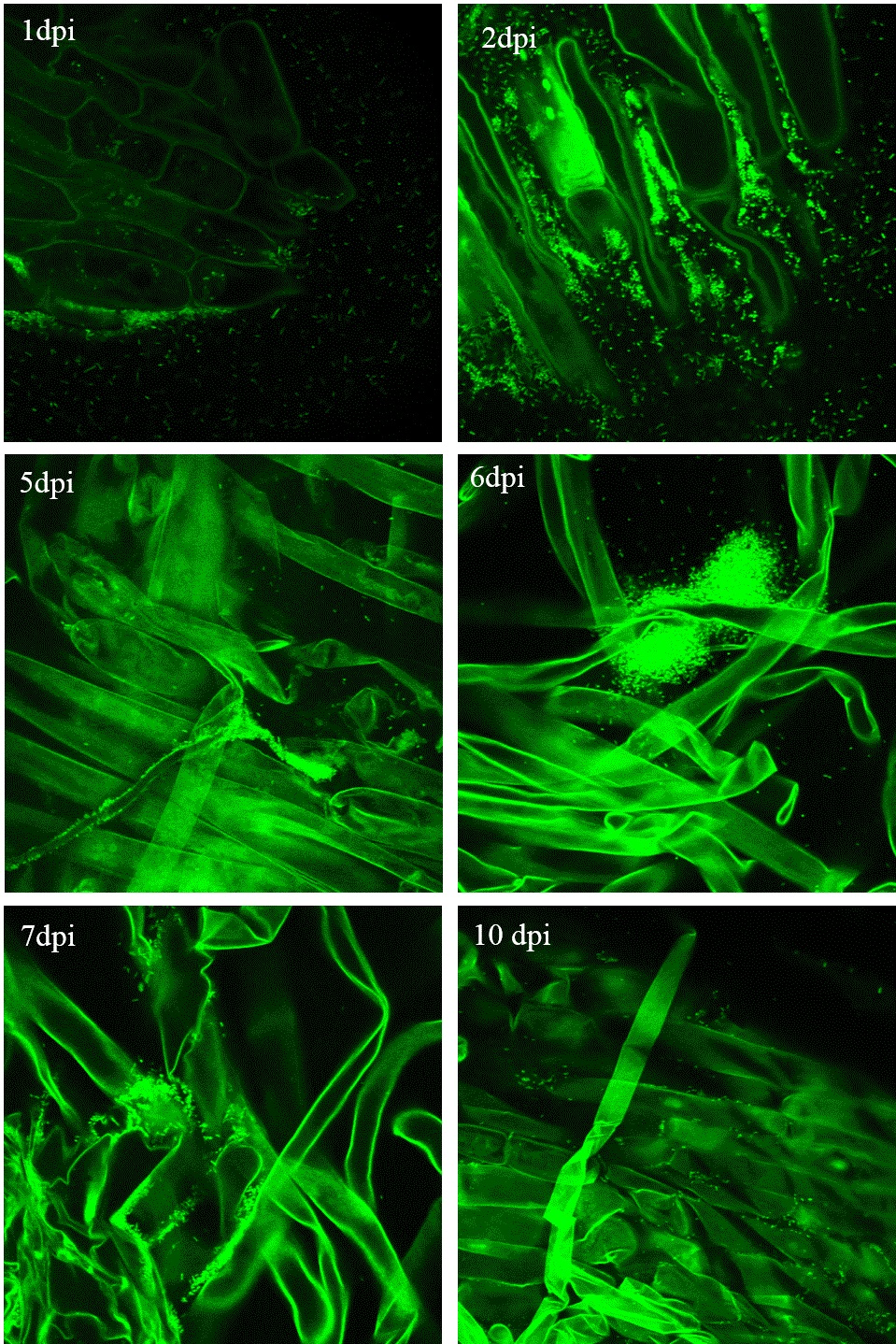

Supplement: Figure S2 — Colonization of barley roots by A. radicis N35 GFP in a monoxenic system at different time points after inoculation (dpi as indicated in the pictures). [file Image2.JPEG]

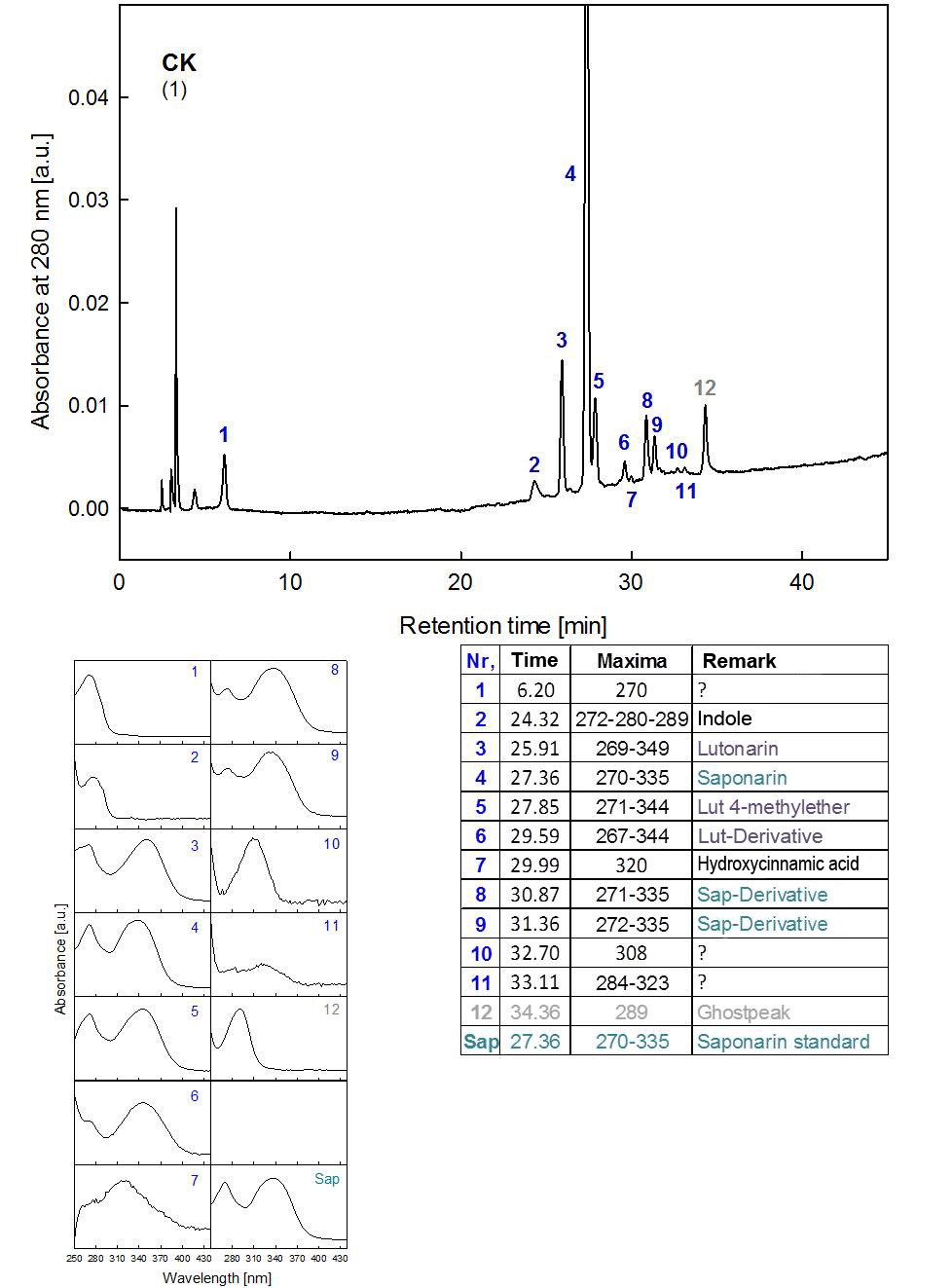

Supplement: Figure S3 — Upper part: HPLC-separation of compounds with absorbance at 280 nm (see MM). Lower part: UV-spectra of the different eluted compounds. [file Image3.JPEG]

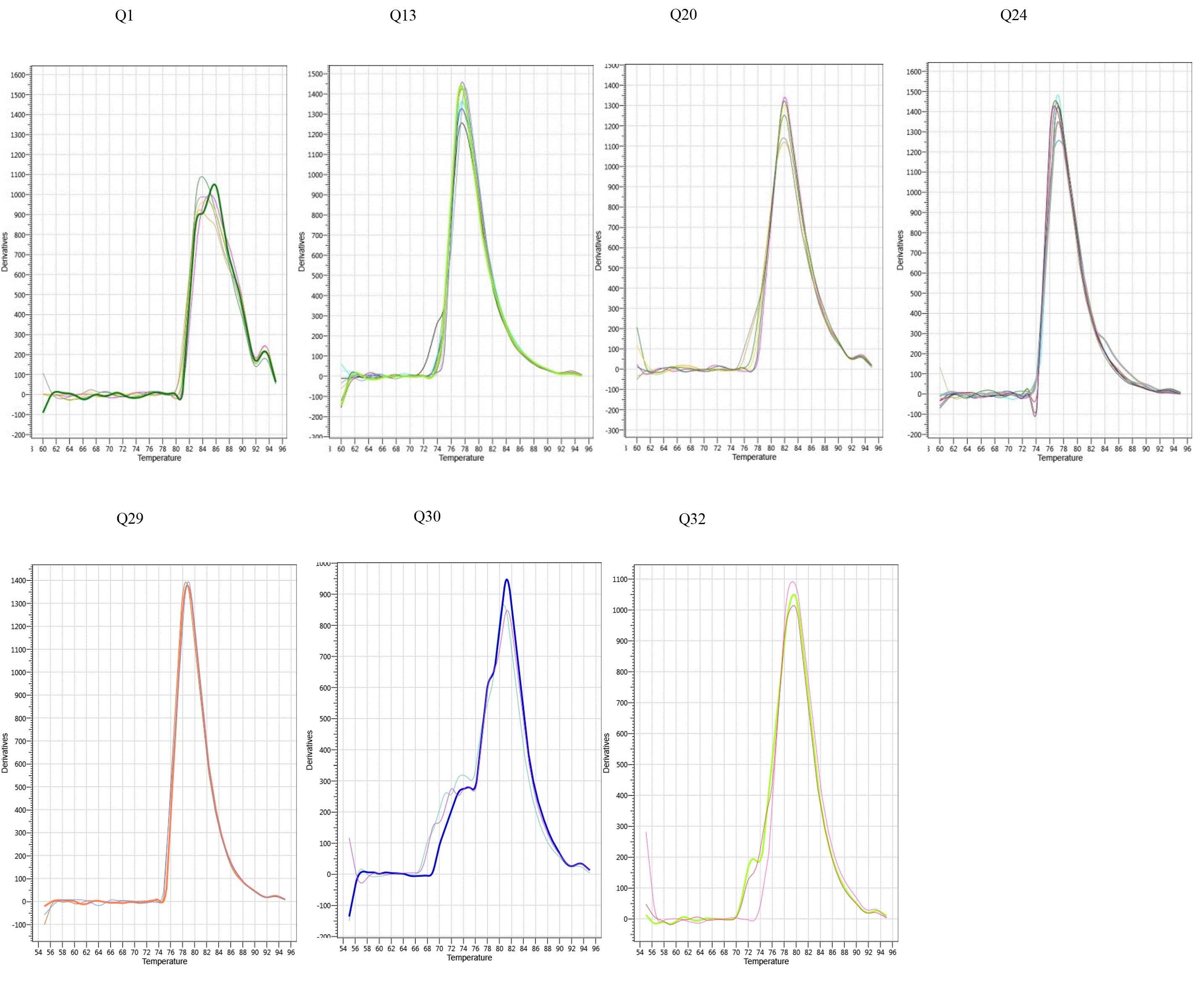

Supplement: Figure S4 — qPCR primer melting curves. [file Image4.JPEG]
